# Belastungsinduzierte ST-Strecken-Hebungen und Kammerflimmern

**DOI:** 10.1007/s00063-025-01346-w

**Published:** 2025-11-18

**Authors:** Sascha Macherey-Meyer, Daniel Steven, Maria Isabel Koerber

**Affiliations:** Medizinische Fakultät und Uniklinik Köln, Klinik III für Innere Medizin, Universität zu Köln, Kerpener Straße 62, 50937 Köln, Deutschland

**Keywords:** Akutes Koronarsyndrom, Koronare Herzerkrankung, ANOCA, Vasospastische Angina, Herzstillstand, Acute coronary syndrome, Coronary artery disease, ANOCA, Vasospastic angina, Cardiac arrest

## Fallpräsentation

Ein 44-Jähriger stellt sich zur Verlaufskontrolle nach Implantation eines subkutanen internen Kardioverter-Defibrillators (S-ICD) in der kardiologischen Sprechstunde vor. Die Holter-Analyse im Rahmen der S‑ICD-Kontrolle zeigt mehrere adäquate Schockabgaben bei Kammerflimmern zwei Tage zuvor. Gezielt befragt, berichtet der Patient, dass er während des Treppensteigens ein Thoraxschmerzereignis begleitet von einer Präsynkope erlitten hat. Diese Symptomatik korrelierte zeitlich mit den Schockabgaben.

Aus der medizinischen Vorgeschichte ist zu erheben, dass der S‑ICD einen Monat zuvor in Folge eines überlebten Herz-Kreislauf-Stillstands mit schockbarem Primärrhythmus implantiert worden ist. Der Herzstillstand ereignete sich beim Fahrradfahren. Das damalige 12-Kanal-Elektrokardiogramm (EKG) nach Rückkehr des Spontankreislaufs ergab keinen Hinweis auf einen akuten Koronararterienverschluss. Die selektiv durchgeführte Koronarangiographie schloss eine obstruktive koronare Makroangiopathie aus. Eine ergänzte kardiale Magnetresonanztomographie ergab korrespondierend keinen Hinweis auf eine zugrunde liegende ischämische Herzerkrankung – insbesondere zeigte sich keine Infarktnarbe. Weiterhin bestanden keine Zeichen einer Myokarditis oder einer spezifischen Kardiomyopathie. Die linksventrikuläre systolische Funktion war mit einer Ejektionsfraktion von 48 % leichtgradig reduziert. Bis zu diesem Akutereignis gab es mit Ausnahme einer adäquat eingestellten arteriellen Hypertonie keinerlei chronische Erkrankungen. Der Patient war aktiver Raucher. Er berichtete von einem gelegentlichen Tetrahydrocannabinol-Konsum. In der Familienanamnese gab es keinen Fall eines plötzlichen Herztods.

**Abb. 1:**
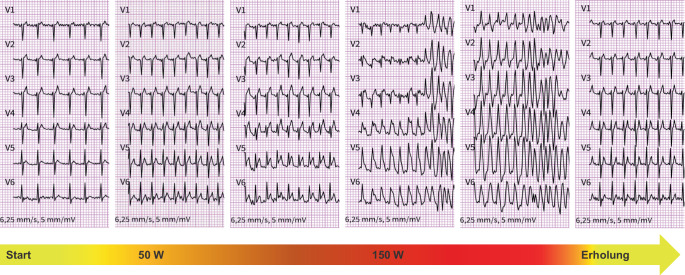
Belastungs-EKG-Ausschriebe. Die Abbildung zeigt ausschnittsweise die Brustwandableitungen während einer Belastungs-EKG-Untersuchung mit schrittweiser Erhöhung der Intensität. (*W* Watt)

Rückblickend hatten diese wiederholten Episoden von Kammerflimmern leichte körperliche Beanspruchung als verbindendes Element und somit als mögliche Ursache. Um dieser Vermutung nachzugehen, wurde eine Belastungs-EKG-Untersuchung durchgeführt. Die kontinuierliche Aufzeichnung zeigte mit zunehmender Belastungsintensität dynamische EKG-Veränderungen (Abb. 1).

## Wie lautet Ihre Diagnose?

## Wie ist der EKG-Ausschrieb zu bewerten?

### Interpretation, klinischer Verlauf und Diskussion

Der Patient belastete sich bis 150 Watt, während der Untersuchung war der S‑ICD deaktiviert. Hierunter kam es zunächst zu einem adäquaten Anstieg der Herzfrequenz sowie zu einer pathologischen Verbreiterung des QRS-Komplexes. Begleitend entwickelte der Patient ST-Strecken-Hebungen in den Vorderwandableitungen. Plötzlich kam es zu einer ventrikulären Tachykardie, die in Kammerflimmern degenerierte. Zu diesem Zeitpunkt beklagte der Patient Thoraxschmerz und es kam zu einer Präsynkope. Mit dem damit verbundenen Abbruch der Belastung terminierte spontan auch das Kammerflimmern. In der Erholungsphase konnte dann ein Rückgang der anterioren ST-Strecken-Hebungen nachvollzogen werden, währenddessen waren aber noch hochamplitudige T‑Wellen nachweisbar.

**Abb. 2:**
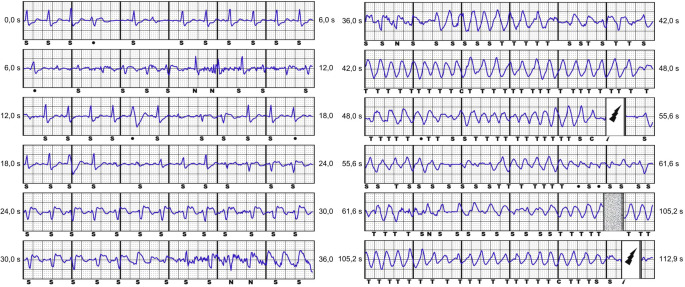
S‑ICD-Holter-Episode. Die Abbildung zeigt die gespeicherte Episode einer S‑ICD-Schockabgabe. Zunächst bestehen im Sinusrhythmus schmale QRS-Komplexe, die sich zunehmend verbreitern. Dann kommt es zur Entwicklung von ST-Strecken-Hebungen. Der Patient entwickelt zuletzt Kammerflimmern, das durch zweimalige Schockabgabe (*Blitzsymbol*) zunächst unterbrochen und schließlich terminiert werden kann. Die Buchstaben S, C, N, T sind Annotationsmarker des S‑ICD

Die Abfolge von dynamischen ST-Strecken-Hebungen gefolgt von Kammerflimmern konnte gleichsam im Holter-Ausschrieb des S‑ICD mehrere Tage zuvor nachvollzogen werden (Abb. 2).

## Was sind mögliche Ursachen von belastungsinduziertem Kammerflimmern?

Belastungsinduzierte Kammerarrhythmien lassen typischerweise an eine katecholaminerge polymorphe ventrikuläre Tachykardie denken. Der EKG-Befund war hierzu jedoch nicht passend. Stattdessen sprach diese Sequenz von ischämietypischem EKG-Befund im Kontext des Thoraxschmerzes für eine transiente Koronarischämie, unter Kenntnis der Vorbefunde wurde die Arbeitsdiagnose „angina pectoris with non-obstructive coronary arteries“ (ANOCA) gestellt [1, 2].

**Diagnose:** „angina pectoris with non-obstructive coronary arteries“ (ANOCA)

### Infobox „Angina pectoris with non-obstructive coronary arteries“ (ANOCA)

- ANOCA ist eine Arbeitsdiagnose.
- Man spricht von ANOCA, wenn klinisch eine Angina pectoris/ein Anginaäquivalent vorliegt und gleichzeitig eine obstruktive koronare Herzerkrankung ausgeschlossen wird.
- Zugrunde liegende Pathologien umfassen u. a. eine mikrovaskuläre Dysfunktion, mikrovaskuläre Vasospasmen, endotheliale Dysfunktion, epikardiale Vasospasmen oder Myokardbrücken.
- Eine funktionelle Koronardiagnostik inkl. Provokationstestung sowie eine intrakoronare Bildgebung sind diagnostische Werkzeuge zur weiteren Einordnung.

**Abb. 3:**
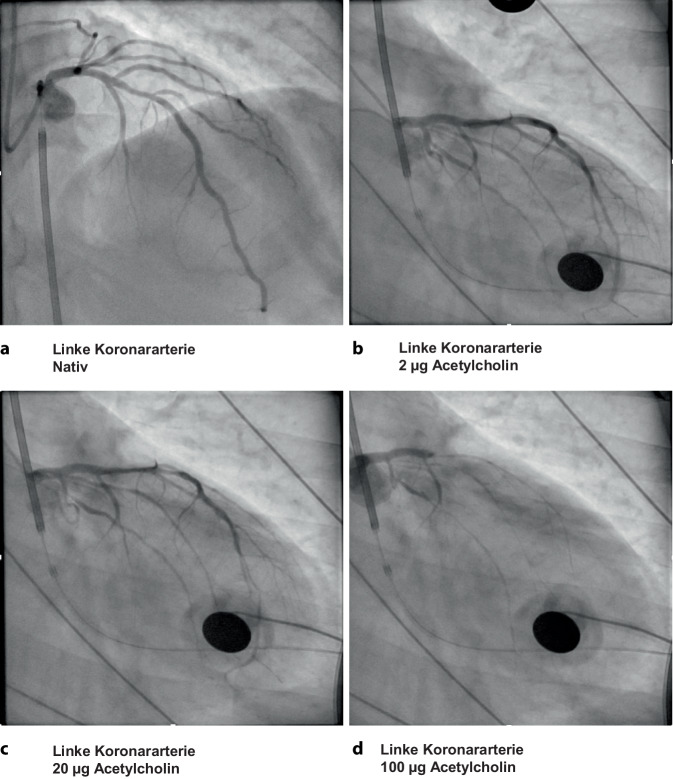
Koronarangiographie und Acetylcholintestung. **a** Native Darstellung der linken Koronararterie. **b** Darstellung der linken Koronararterie nach intrakoronarer Applikation von 2 µg Acetylcholin. Es zeigt sich eine diskrete Gefäßverschmälerung im mittleren Segment. **c** Darstellung der linken Koronararterie nach intrakoronarer Applikation von 20 µg Acetylcholin. Es zeigt sich eine fortgeschrittene Gefäßverschmälerung betont im mittleren Segment. **d** Darstellung der linken Koronararterie nach intrakoronarer Applikation von 100 µg Acetylcholin. Es zeigt sich nahezu eine Totalokklusion

Zur weiteren Abklärung dieser Arbeitsdiagnose wurde eine funktionelle Koronararteriendiagnostik mit Acetylcholintestung indiziert [1, 3]. Die Koronarangiographie schloss erneut relevante Koronarstenosen unter Nativbedingungen aus (Abb. 3a). Es erfolgte dann die schrittweise intrakoronare Applikation von Acetylcholinboli (2 µg, 20 µg, 100 µg). Hierunter entwickelte der Patient Thoraxschmerz, der vergleichbar war zu den vorherigen Episoden. Dosisabhängig kam es dann zu dynamischen ST-Strecken- und T‑Wellen-Veränderungen, die von einem progressiven epikardialen Vasospasmus der Vorderwandarterie begleitet wurden (Abb. 3b, c). Letztlich resultierte die Applikation von 100 µg Acetylcholin in einer Verschmälerung der Vorderwandarterie > 90 % des Ausgangsdiameters, es kam fast zur Totalokklusion (Abb. 3d). Nach zügiger intrakoronarer Applikation von Glyceroltrinitrat konnte der Vasospasmus aufgehoben werden, hierunter nahm dann auch der Thoraxschmerz umgehend ab. Der so provozierte Vasospasmus war mit diesem Befund nach Acetylcholintestung deutlich von einer endothelialen Dysfunktion abzugrenzen und in Einklang zu bringen mit der Diagnose einer vasospastischen Angina pectoris [2].

Die vasospastische Angina tritt typischerweise in Ruhebedingungen oder bei emotionaler Beanspruchung auf [2–4]. Die Diagnose kann in Unklarheit bezüglich der Trigger bzw. angesichts des unspezifischen Charakters leicht verpasst werden, so wie es auch nach dem ersten Ereignis mit plötzlichem, überlebten Herztod im geschilderten Patientenfall trotz fachgerechter, multimodaler Diagnostik geschah. Dies unterstreicht die Bedeutung einer sorgfältigen Anamnese und einer funktionellen Koronardiagnostik, falls eine nichtobstruktive Koronarischämie vermutet wird [1, 2]. Der Trigger im vorliegenden Fall war geringe körperliche Aktivität. Bis dato sind nur wenige Berichte veröffentlicht, die ordinäre Aktivität mit epikardialen Vasospasmen direkt in Verbindung bringen. Die genaue Prävalenz bleibt daher unklar.

Die Sequenz aus Koronarspasmus gefolgt von malignen Arrhythmien während einer Belastungs-EKG-Untersuchung wurde bereits 1975 beschrieben [5]. Die Untersuchungsmodalität ist jedoch durch funktionelle Bildgebung zunehmend in den Hintergrund gerückt. In der Diagnostik der belastungsinduzierten vasospastischen Angina pectoris bietet das flächendeckend verfügbare Belastungs-EKG indes einen Informationsgewinn und kann hilfreich sein, diesen ANOCA-Subtyp zu erkennen.

Die vasospastische Angina pectoris ist eine prognoselimitierende Erkrankung mit erhöhtem Risiko für kardiale Ereignisse [2]. Im vorgestellten Fall kam es sogar zum plötzlichen Herztod bei Kammerflimmern, der durch fachgerechte Therapie überlebt worden ist. Eine konsequente Therapie war daher indiziert. Nikotin ist ein bekannter Stimulus [4], eine strikte Karenz wurde dem Patienten empfohlen. Weiterhin wurde eine vorbestehende Betablockertherapie beendet und durch eine vasodilatierende Behandlung mittels Kalziumkanalblocker ersetzt. Während der Aufdosierungsphase des Kalziumkanalblockers wurde begleitend ein lang wirksames Nitrat verschrieben. Unter dieser Therapie kam es während der ersten 30 Tage zu keiner Rezidivepisode.

## Fazit für die Praxis

- Die vasospastische Angina pectoris kann in Einzelfällen auch durch Alltagsaktivitäten und leichte Belastung provoziert werden.
- Das Belastungs-EKG kann ein sinnvolles Instrument sein, wenn körperliche Aktivität als Trigger vermutet wird.
- Eine funktionelle Koronardiagnostik einschließlich Acetylcholintestung ist zur Diagnosestellung erforderlich.
- Die Erstlinientherapie besteht aus Kalziumkanalblockern, eine Kombinationsbehandlung mit lang wirksamen Nitraten kann erwogen werden.

## Data Availability

Entfällt.
